# Contact with Counter-Stereotypical Women Predicts Less Sexism, Less Rape Myth Acceptance, Less Intention to Rape (in Men) and Less Projected Enjoyment of Rape (in Women)

**DOI:** 10.1007/s11199-016-0679-x

**Published:** 2016-09-30

**Authors:** Miriam Taschler, Keon West

**Affiliations:** Department of Psychology, Goldsmiths, University of London, New Cross Road, New Cross, London, SE14 6NW UK

**Keywords:** Contact hypothesis, Sexism, Sexual violence, Violence against women, Rape, Rape myth acceptance

## Abstract

Intergroup contact—(positive) interactions with people from different social groups—is a widely researched and strongly supported prejudice-reducing mechanism shown to reduce prejudice against a wide variety of outgroups. However, no known previous research has investigated whether intergroup contact can also reduce sexism against women. Sexism has an array of negative outcomes. One of the most detrimental and violent ones is rape, which is both justified and downplayed by rape myth acceptance. We hypothesised that more frequent, higher quality contact with counter-stereotypical women would predict lower levels of sexism and thus less rape myth acceptance (in men) and less sexualised projected responses to rape (in women). Two studies using online surveys with community samples supported these hypotheses. In Study 1, 170 male participants who experienced more positive contact with counter-stereotypical women reported less intention to rape. Similarly, in Study 2, 280 female participants who experienced more positive contact with counter-stereotypical women reported less projected sexual arousal at the thought of being raped. Thus, the present research is the first known to show that contact could be a potential tool to combat sexism, rape myth acceptance, intentions to rape in men, and sexualisation of rape by women.

*Intergroup contact*—(positive) interactions between members of different social groups—is an extensively tested and widely used way of reducing intergroup prejudice (Allport 1954; Oskamp and Jones 2000). It reduces intergroup bias and anxiety, improves intergroup relations, and increases trust between members of different groups (Pettigrew and Tropp 2008). These effects occur across a wide range of outgroup targets and across varied research settings (Eller et al. 2007; Evans-Lacko et al. 2013; King et al. 2009; Voci and Hewstone 2003; West and Hewstone 2012; West et al. 2014). Moreover, the attitudes generated through intergroup contact tend to generalise to the whole outgroup rather than applying solely to an individual target member (Pettigrew and Tropp 2006). Although intergroup contact has been found to work in a variety of settings, Allport (1954) describes conditions under which intergroup contact is optimized—namely, equal status; common goals; intergroup cooperation; and institutional support from the law, authorities, and customs. These conditions are not necessary; intergroup contact can, and often does, still have positive effects in their absence. Rather, these conditions facilitate the reduction of prejudice with intergroup contact and represent the optimal conditions under which contact should occur (Brown and Hewstone 2005; Pettigrew and Tropp 2006).

Over 60 years of prior research, and over 500 individual studies, have investigated the effects of intergroup contact on prejudice, including prejudice based on ethnicity, sexual orientation, physical disability, neurocognitive deficits, mental health, and age (see Pettigrew and Tropp 2006 for a review). However, despite the vast and varied nature of this prior body of research, we are unaware of any prior research that investigated the effects of contact on sexism. This oversight may be due to a number of reasons. Men and women co-exist in roughly equal numbers in every society in the world (United Nations Department of Economic and Social Affairs 2015). Moreover, men and women generally have a large number of cross-gender relationships in both their social and professional lives (Becker et al. 2014). Therefore, prior researchers may have considered contact to be ubiquitous, or at least too common to be a useful predictor of cross-gender attitudes. Nonetheless, this perspective can be challenged. Despite the co-existence of men and women in all societies, sexism remains a significant global problem (Bunch 1990), with implications for employment (Bhatnagar and Swamy 1995), childcare (Caplan and Hall-McCorquodale 1985), and even physical safety (Forbes et al. 2004).

Even in relatively egalitarian societies, subtle and implicit sexism continues to play a meaningful role, affecting women’s abilities to achieve equal status (Glick and Fiske 2001). Similarly, although men and women frequently interact across genders, these interactions may not always occur under intergroup contact’s optimal conditions of equal status, common goals, cooperation, and institutional support. In particular, women are still widely considered subordinate to men (Ridgeway 2001; Whyte 1978) and frequently occupy lower status roles (Office for National Statistics 2013). Thus, although cross-gender contact is frequent, it may nonetheless be unusual for men to interact with women who are of equal or higher status than themselves. Therefore, this type of intergroup contact may be a particularly meaningful predictor of sexist attitudes. Satisfying Allport’s (1954) condition of equal status, the present study focuses on contact with counter-stereotypical, high-status women (i.e., women in positions of power or authority) and its influence on sexism.

## Sexism and Men’s Intentions to Rape

Sexist prejudice has been described as ambivalent prejudice against women rather than as uniform antipathy (Glick and Fiske 1996). This multidimensional construct includes both hostile and benevolent sexism. *Hostile sexism* is a manifestation of the classical definition of prejudice with antipathy and hostility towards women. In contrast, *benevolent sexism* is characterized by sexist attitudes that limit women to stereotypical roles but are subjectively positive and affectionate towards women. Sexism has been linked to a variety of negative outcomes, including rape myth acceptance (Glick and Fiske 1996; Viki et al. 2004), which in turn has been linked to sexual assault and, at its worst, with rape (Koss 1985; Koss and Dinero 1989; Malamuth et al. 1991).

We chose rape as our outcome measure because it is one of the most profound and violent affirmations of sexism. Rape myths are also a matter of heated and wide-spread contemporary discussion. For example, the recent case of Brock Turner—an American college student who was jailed for only 6 months after raping a fellow student—sparked national and international debates on sexism, rape, and rape myths, as well as the prosecution of rapists (Carroll 2016; Stack 2016).

Rape has been described as a “logical and psychological extension of a dominant – submissive, competitive, sex role stereotyped culture” (Burt 1980, p. 229). In the United Kingdom alone, about 85,000 women report being raped each year (Ministry of Justice et al. 2013). Rape has widespread consequences for the victim’s psychological and physical health, including an increased likelihood of post-traumatic stress disorder (PTSD), depression, anxiety, heightened substance abuse, and suicidal ideation (Resick 1993). Thus, it has been described as one of the most severe of all traumas (Campbell et al. 2009). Research has shown that those effects are not only present in the short term, but also can affect the victim decades after the trauma takes place (Sachs-Ericsson et al. 2014).

Sexism enforces and maintains a male-dominated power structure (Glick and Fiske 1996), and hostile sexism specifically is closely related to a violent outlet of the sexist belief. Therefore, hostile sexism can be a major motivation for rape, which can be seen as a physical manifestation of sexist prejudice. Rape can then be justified, and its seriousness denied, through beliefs in rape myths (Lonsway and Fitzgerald 1994). *Rape myths* are stereotypical beliefs that are used to shift the burden of responsibility for rape from the perpetrator onto the victim (Burt 1980). Because they both deny and justify sexual violence in men (Lonsway and Fitzgerald 1994), endorsement of rape myths may lead to a heightened chance of committing rape (Edwards et al. 2014; Koss 1985; Koss and Dinero 1989; Malamuth et al. 1991). Reducing sexist attitudes should have meaningful positive consequences, lowering rape myth acceptance, and reducing men’s willingness to rape women.

## Sexism and Women’s Responses to Rape

Although men are more frequently perpetrators of rape and other sexual violence, not only men ascribe to sexism. Women, like all members of devalued groups, can absorb and internalize negative messages about themselves (Nosek et al. 2002), tailoring their expectations of themselves accordingly (Irving and Hudley 2008). As such, women, like men can hold sexist beliefs *against women*. This may predict a variety of counterproductive responses such as rape myth acceptance and its related negative psychological and behavioural effects (Frese et al. 2004). Women may use rape myths to deny their own vulnerability (Lonsway and Fitzgerald 1994); if a woman believes that only women who dress provocatively or behave promiscuously get raped, she can feel protected from the possibility of being raped by avoiding these behaviours.

Moreover, if a woman is raped, rape myth acceptance may lead the victim to blame herself for the assault (Frese et al. 2004). This self-blame has widespread implications for the victim’s recovery; trauma-related guilt has been linked with PTSD, depression, negative self-esteem, shame, social anxiety, and suicidal thoughts (Kubany and Watson 2003). There is also a reduced likelihood of reporting the crime if the victim blames herself for the incident (Frese et al. 2004). Indeed estimates suggests that 89 % of rape incidences in the United Kingdom are never reported (Ministry of Justice et al. 2013). Rape myths also reduce the likelihood of investigating the crime, which leads to reduced punishment for perpetrators and, in turn, to a heightened likelihood of raping again (Frese et al. 2004). As a result, the victim may feel, and actually be, even more helpless about being raped.

One rape myth of particular interest to the present study is the perception that rape is a sexual act rather than a violent one (Emmers-Sommer et al. 2006). This belief can lead some women to project sexual arousal at the thought of being raped, which further downplays the seriousness of rape. Thus, we measure projected sexual arousal in women in our second study. Because of previous research linking sexism to rape myth acceptance (Glick and Fiske 1996; Viki et al. 2004), we expect positive relationships among sexism, rape myth acceptance, and projected sexual arousal at the thought of being raped in women.

## The Current Research

The current research addresses a gap in the literature by being the first known to investigate whether direct contact with counter-stereotypical women predicts less sexism against women, specifically less hostile and benevolent sexism, lower rape myth acceptance, less intention to rape (in men), and less projected sexual arousal at the thought of being raped (in women). We thus investigated the effects of both intergroup contact and intragroup contact. Study 1 recruited male participants and investigated the relationships among positive contact with counter-stereotypical women, sexism, rape myth acceptance, and intention to rape. Study 2 sampled female participants and investigated the relationships among positive contact with counter-stereotypical women, sexism, rape myth acceptance, and projected sexual arousal.

## Study 1

Using a correlational design, we investigated whether male participants’ self-reported prior contact with counter-stereotypical women would predict less intention to rape and whether that relationship was mediated by hostile sexism, benevolent sexism, and rape myth acceptance. We expected contact to be negatively associated with both hostile and benevolent sexism, as well as rape myth acceptance and thus intention to rape.

### Method

#### Participants and Design

Participants were 170 male British members of the general public (*M*_age_ = 25.44, *SD* = 8.56, range = 18–65) who completed a questionnaire about their contact with counter-stereotypical women, sexism, rape myth acceptance, and intention to rape women. Participants were mostly White (104, 61.2 %) with small numbers of other ethnicities: 19 (11.2 %) Black, 7 (4.1 %) East Asian, 7 (4.1 %) South Asian, and 33 (19.4 %) other. Most participants were also either non-religious (74, 43.5 %) or Christian (63, 37.1 %) with small proportions belonging to various other religions (33, 19.4 %). After completing the survey participants were thanked for their time and debriefed. No monetary compensation was offered in exchange for completing the study. This study was approved by the relevant university ethics committee, which adhered to the guidelines of the British Psychological Society.

#### Procedure

Participants were recruited online, via word-of-mouth, and through Internet forums to complete an online survey ostensibly about “sex and sexual attractiveness.” Filler items were included throughout the study to distract participants from the true hypotheses. In keeping with the cover story, these filler items asked participants to report the likelihood that they would try a number of sexual behaviours including oral sex, anal sex, homosexual sex, group sex, bondage, whipping, spanking, cross-dressing, and paedophilia. With the exception of the filler items, all measures that were used in this current research are well-established scales that have been validated by prior research and widely used thereafter.

The order of presentation of all measures was counter-balanced for each participant so that each scale had an equal likelihood of being presented to participants first. There was no indication that any participants dropped out of the study because of the questions related to sexual behaviours (e.g., rape, bongage, paedophilia). Indeed, all the participants completed these measures, and none of the participants completed the contact measures but declined to complete the measures concerning sexual behaviours. Of the 195 participants who started the survey, the 25 who did not finish the survey completed the items about sexual behaviours but did not complete the items measuring contact. There was also no evidence that this attrition was systematic. A series of independent samples *t*-tests revealed that participants who did complete the study did not differ from those who did not complete the study on any of the measures of sexism, rape myth acceptance, or sexual behavioural intentions (1.71 < *t* < .23; .82 < *p* < .09).

#### Contact With Counter-Stereotypical Women

To measure quantity of contact with counter-stereotypical, high-status women we adapted five items from Turner et al. (2007). Participants responded to each of the following questions about their experiences with “women in positions of power or authority, or women who are more senior than you occupationally”: “Right now, how many of your close friends are women like these?” (1 = *none*, 2 = *a few*, 3 = *about half*, 4 = *most*, 5 = *almost all*); “Right now, how many of the people you see on a typical day are women like these?” (1 = *none*, 2 = *a few*, 3 = *about half*, 4 = *most*, 5 = *almost all*); “How often do you spend time with friends who are women like these?” (1 = *never*, 2 = *occasionally*, 3 = *sometimes*, 4 = *quite a lot*, 5 = *all the time*); “How often do you spend time with co-workers or fellow students who are women like these?” (1 = *never*, 2 = *occasionally*, 3 = *sometimes*, 4 = *quite a lot*, 5 = *all the time*); and “How many of the people you work or study with are women like these?” (1 = *none*, 2 = *one*, 3 = *two to five*, 4 = *five to ten*, 5 = *more than ten*). Ratings across these five items were averaged for each participant (*α* = .88) such that higher scores indicate greater contact.

To measure quality of contact, we included seven items used in prior intergroup contact research (West et al. 2014; West and Hewstone 2012). Participants reported on a 7-point scale ranging from −3 (*not at all*) to +3 (*very*) how pleasant, friendly, negative (reversed), enjoyable, difficult (reversed), cooperative, natural, and superficial (reversed) their contact experiences with these women had been (*α* = .78). Ratings across these seven items were averaged for each participant. Contact Theory stresses the importance of considering quantity and quality of contact simultaneously. Thus, as has been done in prior research (Evans-Lacko et al. 2013; Voci and Hewstone 2003; West et al. 2014; West and Hewstone 2012), we created a single measure of contact by using the product of participants’ mean contact quantity and quality scores. Thus this score could range from −15 to +15, wherein higher scores indicated more frequent and higher-quality contact.

#### Sexism

To assess hostile sexism, we used the 11 hostile sexism items (α = .88) from Glick and Fiske’s (1996) Ambivalent Sexism Inventory. Higher scores indicated more antipathy and hostility towards women (e.g., “Women are too easily offended” and “Women seek to gain power by getting control over men”). To assess benevolent sexism we used the 11 benevolent sexism items (α = .78) from Glick and Fiske’s (1996) ambivalent sexism scale. Higher scores indicated positive but nonetheless stereotypical views of women (e.g., “Many women have a quality of purity that few men possess” and “Women should be cherished and protected by men”). Participants responded to these items using 5-point Likert scales ranging from 1 (*Strongly Disagree*) to 5 (*Strongly Agree*). The ambivalent sexism scale has been widely used in prior research, and it is predictive of awareness of sexism, responses to female authority, and both implicit and explicit forms of sexism (Becker and Swim 2011; Rudman and Kilianski 2000).

#### Rape Myth Acceptance

To assess rape myth acceptance, we used 11 items (α = .82) from Burt's (1980) Rape Myth Acceptance Scale. Higher scores indicate a greater tendency to shift the burden of responsibility for rape from the perpetrator to the victim (e.g., “In the majority of rapes, the victim is promiscuous or has a bad reputation” and “When women go around braless or wearing short skirts and tight tops, they are just asking for trouble.”). Participants responded to these items using 7-point Likert scales from 1 (*Strongly Disagree*) to 7 (*Strongly Agree*). Prior research has found rape myth acceptance to be associated with long-term exposure to sex-stereotyped media, adversarial sexual beliefs, and acceptance of interpersonal violence (Dill et al. 2008; Ryan and Kanjorski 1998).

#### Intention to Rape

Finally, to assess participants’ intention to rape, we used two high face-valid items (*r* = .68, *p* < .001) from the Attraction to Sexual Aggression Scale (Malamuth 1989), which have also been used in subsequent research (Edwards et al. 2014): Using 5-point Likert scales (1 = *not at all likely*, 5 = *very likely*), participants responded to the following statements: “If you could be assured that no one would know and that you could in no way be punished, how likely, if at all, would you be to rape?” and, “If you could be assured that no one would know and that you could in no way be punished, how likely, if at all, would you be to force a female to do something sexual she didn’t want to do?”

### Results

Means and standard deviations as well as correlations of all relevant measures can be found in Table 1. These initial correlations revealed that positive contact was significantly negatively associated with both hostile sexism and rape myth acceptance, but not with benevolent sexism or intentions to rape. Correlations among hostile sexism, benevolent sexism, rape myth acceptance, and intentions to rape were all positive and most (5 of 6) were statistically significant.

**Table 1:** Descriptive statistics and correlations for all relevant variables, study 1 (with men)

Variables	Actual			Correlations						
	Range	*M*	*SD*	1	2	3	4	5	6	7
1. Age	18 – 65	25.44	8.56	--						
2. Contact quantity	1 – 5	2.71	.93	.11	--					
3. Contact quality	−1.86 – 3	1.05	1.09	.06	.33***	--				
4. Contact index	−6.31 – 15	3.16	3.47	.06	.56***	.91***	--			
5. Hostile sexism	1 – 4.64	2.70	.87	-.13	-.18*	-.27***	-.30***	--		
6. Benevolent sexism	1.36 – 4.55	2.86	.75	-.05	.04	-.03	-.05	.57***	--	
7. Rape myth acceptance	1 – 6.45	2.02	.93	-.10	-.18*	-.33***	-.37***	.59***	.41***	--
8. Intention to rape	1 – 5	1.63	1.021	.20**	.03	-.09	-.13	.28***	.07	.35***

*p < .05. **p < .01. ***p < .001

Relationships among variables were investigated with structural equation modelling in AMOS. Because the contact index was a single item (i.e., the product of quality and quantity of contact), this was included as an observed variable. All other multi-item constructs were included as latent variables in the model (Byrne 2001). Scales with large numbers of individual items are cumbersome for structural equation models. Thus the eleven observed items for the hostile sexism, benevolent sexism and rape myth acceptance scales were parcelled into three items for each scale following recommendations concerning parcelling with well-established, unidimensional constructs (Cheung and Chan 2002; Little et al. 2002; MacCallum and Austin 2000).

We tested an initial model in which contact predicted both hostile sexism and benevolent sexism, which in turn predicted rape myth acceptance, which in turn predicted intention to rape. We assessed the goodness-of-fit of the model, and alternative models, by using the Chi-square test (*χ* ^2^ ideally should be non-significant, but should not be weighted too much for sample sizes around 200 or more; cf., Hooper et al. 2008), Chi-square/degree of freedom ratio (*χ*2/df should be less than 5, ideally less than 3; Hooper et al. 2008), the Comparative Fit Index-CFI (should be greater than .90; cf. Hu and Bentler 1999), the Incremental Fit Index-IFI (should exceed .90; cf. Marsh et al. 2004), the Tucker Lewis Index-TLI (should exceed .90; cf. Hu and Bentler 1999), and the Root Mean Square Error of Approximation-RMSEA (should be less than .10, ideally less than .08; cf. Hu and Bentler 1999; MacCallum et al. 1996).

This initial model fit the data poorly, *χ*2(47) = 147.26, *p* < .001, *χ*2/df = 3.13; CFI = .91; IFI = .91; TLI = .88; RMSEA = .11. Examination of specific pathways in the model showed that benevolent sexism did predict rape myth acceptance (*b* = .25, *p* = .008), and intention to rape (*b* = −.18, *p* = .04), but that contact did not predict benevolent sexism (*b* = −.02, *p* = .34). We thus removed benevolent sexism from the model and tested an alternative model in which contact predicted hostile sexism, which in turn predicted rape myth acceptance, which in turn predicted intention to rape.

**Fig. 1:**
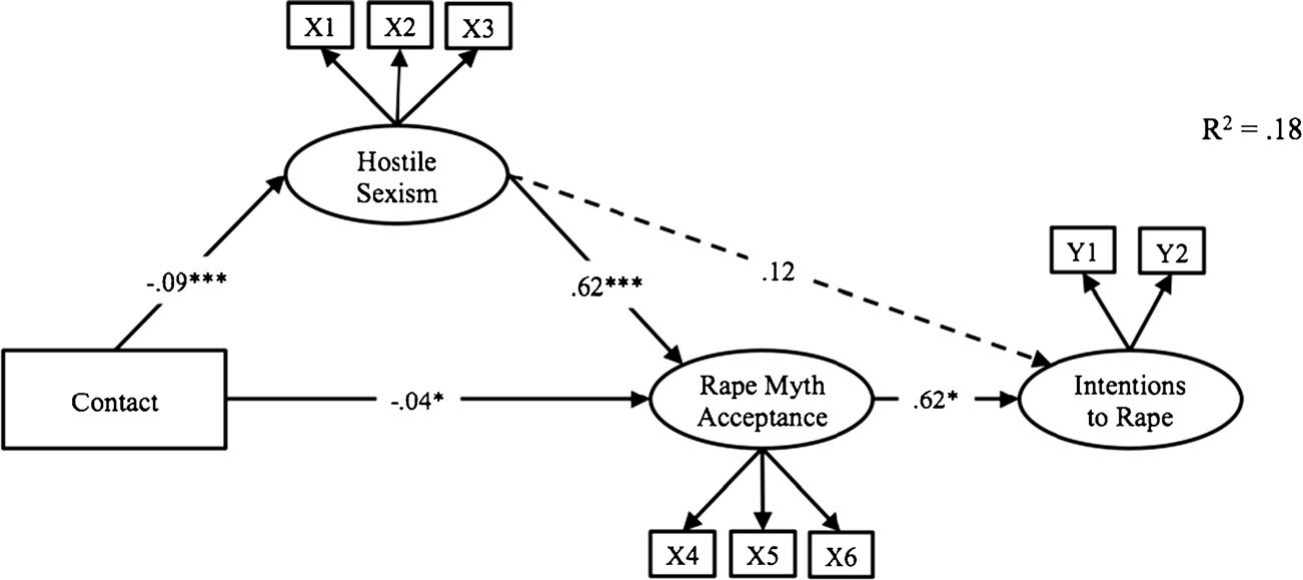
Relationship between contact and intention to rape in men, mediated by hostile sexism and rape myth acceptance. *p < .05. **p < .01. ***p < .001

This model fit the data well, *χ*2(23) = 43.24, *p* = .006, *χ*2/df = 1.88; CFI = .98; IFI = .98; TLI = .96; RMSEA = .07, accounting for 18 % of the variance in intention to rape (see Fig. 1). As we hypothesized, contact predicted less hostile sexism (*b* = −.09, *p* < .001) and less rape myth acceptance (*b* = −.04, *p* = .019). Hostile sexism predicted more rape myth acceptance (*b* = .62, *p* < .001) but not more intention to rape (*b* = .12, *p* = .15). Rape myth acceptance predicted more intention to rape (*b* = .22, *p* = .016). The overall indirect effect of contact on intention to rape was negative (*b* = −.03).

### Discussion

Study 1 investigated the relationships between men’s positive intergroup contact with counter-stereotypical women, sexism, rape myth acceptance and intention to rape. We found that contact indirectly predicted less intention to rape in men, a relationship that was mediated by less hostile sexism and less rape myth acceptance. Benevolent sexism predicted more rape myth acceptance, but (directly) less intention to rape. However, contact did not predict benevolent sexism. The present study is a first indication that contact with counter-stereotypical women may be a useful tool to reduce men’s hostile sexism, rape myth acceptance and willingness to rape. It also highlighted the possibly divergent effects of contact on hostile sexism compared to benevolent sexism.

## Study 2

As mentioned previously, women, like men, can hold sexist beliefs against women, including beliefs endorsing rape myth acceptance. Rape myths shift the responsibility for rape onto (female) victims by suggesting that women are sometimes complicit in getting raped, or that they derive sexual pleasure from being raped (Burt 1980). This bolsters the misconception that rape is a sexual act rather than a violent one. Women who accept these beliefs may downplay the seriousness of rape and imagine the experience as potentially pleasurable or sexually arousing (Emmers-Sommer et al. 2006). However, if positive *intragroup* contact has effects similar to those of *intergroup* contact, we can expect positive contact with counter-stereotypical women to undermine these beliefs in women as well as in men. In Study 2 we thus hypothesised that positive contact with counter-stereotypical women should predict less hostile sexism, less rape myth acceptance, and lower projected sexualisation of rape among women. As in Study 1, relationships among all these variables and benevolent sexism were also investigated.

### Method

#### Participants and Design

Participants were 280 female British members of the general public (*M*_age_ = 23.59, *SD* = 8.11, range = 15–67) who completed a questionnaire about their contact with counter-stereotypical women, sexism, rape myth acceptance, and projected sexual arousal at the thought of being raped. Participants were mostly White (172, 61.4 %) with small numbers of other ethnicities: 34 (12.1 %) Black, 8 (2.9 %) East Asian, 12 (4.3 %) South Asian, and 54 (19.3 %) other. Most participants were also either non-religious (119, 42.5 %) or Christian (118, 42.1 %) with a small proportion belonging to various other religions (43, 15.4 %). After completing the survey participants were thanked for their time and debriefed. No monetary compensation was offered in exchange for completing the study. Ethical approval for the present study was obtained from the relevant university ethics committee.

#### Procedure and Measures

Similar to Study 1, participants were recruited online, via word-of-mouth, and through Internet forums to complete a survey ostensibly about “sex and sexual attractiveness.” Filler items were included throughout the study to distract participants from the true hypotheses. In keeping with the cover story, these filler items asked participants to report their projected sexual arousal at participating in a number of sexual behaviours including oral sex, anal sex, homosexual sex, group sex, bondage, whipping, spanking, cross-dressing, and paedophilia.

Also as in Study 1, the order of presentation of all measures was counter-balanced for each participant so that each scale had an equal likelihood of being presented to the participants first. There was no indication that any participants dropped out of the study because of the questions related to projected sexual arousal. Again, all the participants completed these measures, and none of the participants completed the contact measures but declined to complete the measures concerning sexual arousal. Of the 292 participants who started the study, the 12 participants who did not finish the study completed the items about sexual arousal but did not complete the items measuring contact. Nor was there evidence that this attrition was systematic. A series of independent-samples *t*-tests revealed that participants who did complete the study did not differ from those who did not complete the study on any of the measures of sexism, rape myth acceptance or projected sexual arousal (1.38 < *t* < .28; .78 < *p* < .17).

We measured quantity of contact with counter-stereotypical, high-status women (*α* = .87), quality of contact with counter-stereotypical women (*α* = .88), hostile sexism (*α* = .88), benevolent sexism (*α* = .79), and rape myth acceptance (*α* = .82) with the same scales used in Study 1. To measure women’s projected sexual arousal we asked them to respond to two items (*r* = .51, *p* < .001) adapted from the Attraction to Sexual Aggression Scale (Malamuth 1989). On a sliding scale from 1 (*not at all aroused*) to 100 (*very aroused*), participants responded to the question “How sexually arousing do you think you would find being *raped by someone else*?”; participants also indicated the percentage of women (0–100 %) whom they thought would find being raped by someone else sexually arousing.

### Results

Means and standard deviations as well as correlations of all relevant measures can be found in Table 2. Similar to Study 1, these initial correlations revealed that positive contact was significantly negatively associated with both hostile sexism and rape myth acceptance, but not with benevolent sexism or intentions to rape. Correlations among hostile sexism, benevolent sexism, rape myth acceptance, and intentions to rape were all positive, and most (4 of 6) were statistically significant.

**Table 2:** Descriptive statistics and correlations for all relevant variables, study 2 (with women)

Variables	Actual range	*M*	*SD*	Correlations						
				1	2	3	4	5	6	7
1. Age	15 – 67	23.59	8.11	--						
2. Contact quantity	1 – 5	2.66	.89	.08	--					
3. Contact quality	−2 – 3	1.07	1.17	.04	.38***	--				
4. Contact index	−4.37 – 14.29	3.25	3.66	.10	.60***	.92***	--			
5. Hostile sexism	1 – 4.82	2.31	.82	.03	-.07	-.21***	-.21***	--		
6. Benevolent sexism	1 – 4.45	2.67	.74	-.004	.009	-.03	-.06	.63***	--	
7. Rape myth acceptance	1 – 6.45	1.66	.82	.02	-.01	-.17**	-.18**	.57***	.41***	--
8. Projected sexual arousal	0 – 50.50	4.35	8.55	-.008	.08	-.10	-.09	.03	.05	.25***

*p < .05. **p < .01. ***p < .001

As in Study 1 we investigated the relationships among these variables using structural equation modeling in AMOS. We first tested an initial model in which contact predicted both hostile sexism and benevolent sexism, which in turn predicted rape myth acceptance, which in turn predicted projected sexual arousal. As before, this initial model fit the data poorly, *χ*2(47) = 213.34, *p* < .001, *χ*2/df = 4.54; CFI = .90; IFI = .90; TLI = .85; RMSEA = .11. Examination of specific pathways in the model showed that contact did not predict benevolent sexism (*b* = −.01, *p* = .46) nor did benevolent sexism predict rape myth acceptance (*b* = .08, *p* = .21) or projected sexual arousal (*b* = −.49, *p* = .75). We thus removed benevolent sexism from the model and tested an alternative model in which contact predicted hostile sexism, which in turn predicted rape myth acceptance, which in turn predicted projected sexual arousal.

**Fig. 2:**
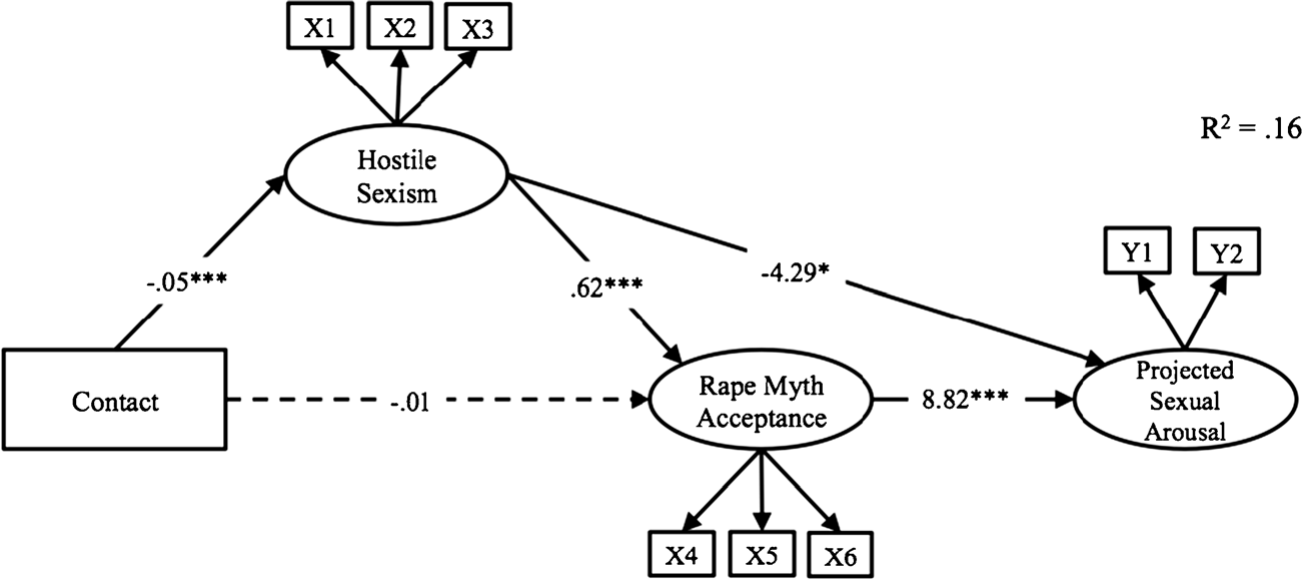
Relationship between contact and projected sexual arousal in women, mediated by hostile sexism and rape myth acceptance. *p < .05. **p < .01. ***p < .001

This model fit the data well, *χ*2(23) = 25.21, *p* = .34, *χ*2/df = 1.10; CFI = 1.00; IFI = 1.00; TLI = 1.00; RMSEA = .019, accounting for 15.8 % of the variance in projected sexual arousal (see Fig. 2). As we hypothesized, contact predicted less hostile sexism (*b* = −.05, *p* < .001), although not (directly) less rape myth acceptance (*b* = −.01, *p* = .26). Hostile sexism predicted more rape myth acceptance (*b* = .62, *p* < .001). Also, although hostile sexism also directly predicted less projected sexual arousal (*b* = −4.29, *p* = .02), as expected, rape myth acceptance predicted more projected sexual arousal (*b* = 8.82, *p* < .001). The overall indirect effect of contact on projected sexual arousal was negative (*b* = −.18).

### Discussion

Study 2 investigated the relationships between intergroup contact with counter-stereotypical women and sexism, rape myth acceptance, and sexual arousal at the thought of being raped among women. We found that positive contact with counter-stereotypical women indirectly predicted less projected sexual arousal at the thought of being raped, a relationship that was mediated by less hostile sexism, which predicted rape myth acceptance, which in turn predicted projected sexual arousal. As in Study 1, benevolent sexism was not an important factor; contact did not predict benevolent sexism, nor did benevolent sexism predict rape myth acceptance or projected sexual arousal. The present study is thus the first known to show that *intragroup* contact with counter-stereotypical women may reduce women’s rape myth acceptance and sexualisation of rape. Our study also highlighted the possibly divergent effects of contact on hostile sexism compared to benevolent sexism.

## General Discussion

In two correlational studies we investigated whether reported contact with counter-stereotypical women would predict less intention to rape in men (Study 1) and less projected sexual arousal at the thought of being raped in women (Study 2), as well as whether these relationships in both cases were mediated by lower levels of sexism and rape myth acceptance. We found support for these hypotheses. In both studies participants who reported more frequent and higher-quality contact also reported less hostile sexism, which in turn predicted less rape myth acceptance, which in turn predicted either less intention to rape (male participants) or less projected sexual arousal at the thought of being raped (female participants). In both studies, benevolent sexism did not contribute meaningfully to the model; contact did not predict benevolent sexism in either study, and in Study 2 (with female participants), benevolent sexism also failed to predict rape myth acceptance. In the following we discuss our findings with reference to study design and implications, limitations, and possible future research.

The current research supports the findings of earlier research on contact (Pettigrew and Tropp 2006). However, it also extends these findings in a number of meaningful ways. It is not a new hypothesis that contact can reduce various forms of prejudice with optimal conditions in place (Brown and Hewstone 2005); several forms of contact and extensions of Contact Theory have demonstrated their usefulness in a wide variety of contexts (Evans-Lacko et al. 2013; Miles and Crisp 2014; Turner et al. 2008; West et al. 2015; West and Turner 2014). However, the present studies are the first known to investigate the contact hypothesis with regards to sexism and some of its most negative manifestations: rape myth acceptance, men’s intentions to rape, and women’s sexualisation of rape. As such, our research has the potential to give rise to an entirely new branch of intergroup contact research that focuses on the benefits of non-stereotypic contact between men and women.

Our research also adds meaningfully to the body of research on sexism, rape, and rape myth acceptance. Prior research has established that hostile and benevolent sexism are independent (although related) constructs that have different effects (Abrams et al. 2003; Becker and Wright 2011; Glick et al. 2000). However, our research suggests another important difference between the two: it appears that hostile sexism is negatively associated with positive contact, but benevolent sexism is not. This difference is possibly because of the overall positive (although restrictive and stereotypical) valence of benevolent sexism. This linkage may make benevolent sexism resistant to interventions like contact, which have been criticised for making cross-group attitudes more positive without necessarily affecting fundamental societal inequalities (Dixon et al. 2010). Further research investigating whether, how, and under what conditions contact affects benevolent sexism could prove very fruitful.

Our research design also incorporated a number of noteworthy strengths. Much social-psychological research, and contact research in particular, has been criticised for the overuse of readily available samples of student participants (Dixon et al. 2005; Henrich et al. 2010; Sears 1986). This practice raises questions about the generalizability of the findings beyond the university setting and for participants with more varied attitudes. The current research, however, benefitted from reasonably large and diverse samples of participants drawn from the general public rather than from student samples.

The design of our research also allows us to counter the possible criticism that our effects were in fact not due to contact, but merely to exposure to counter-stereotypical information (i.e., information about counter-stereotypic women). Prior research shows that counter-stereotypic exemplars can reduce intergroup bias even in the absence of intergroup contact (Blair et al. 2001; Critcher and Risen 2014; West et al. 2011). However, this does not appear to be an adequate explanation for our current results for two reasons. First, our results are in line with prior contact research, which shows that quality of contact plays a vital role (Pettigrew and Tropp 2006; Voci and Hewstone 2003; West and Hewstone 2012). Second, in both studies, *quality* of contact was a stronger (negative) predictor of both hostile sexism and rape myth acceptance than was *quantity* of contact, suggesting that the interactive aspects of contact were more important than the informational aspects in these studies (Pettigrew and Tropp 2008).

### Practice Implications

Concerning practical implications, rape remains a serious problem; approximately 80,000 women and girls in the United States (Amin et al. 2015) and 85,000 women and girls in the United Kingdom (Ministry of Justice et al. 2013) report being raped each year. Furthermore, recent events, such as the trial and sentencing of Brock Turner (Carroll 2016; Stack 2016), reveal that rape myth acceptance and downplaying the seriousness of rape remain serious and widespread social problems. Our research suggests a new potential method of reducing sexism and rape myth acceptance, grounded in a well-established area of social-psychological research—Contact Theory.

Although our research is the first known of its kind and must necessarily be considered preliminary rather than definitive, our findings may eventually be useful in designing and implementing intervention programs to reduce the occurrence of rape and increase the seriousness with which it is regarded. Contact with counter-stereotypic women is a relatively cheap and easy intervention strategy; this would improve its potential applicability and practical feasibility. More broadly, positive contact with counter-stereotypical women throughout society could be used as a means of tackling other widespread manifestations of sexism, such as the pay gap between men and women (Blackaby et al. 2005) or the relatively low number of women in science, technology, engineering and math (STEM) fields (Rosenthal et al. 2011).

### Limitations and Future Research Directions

Our research focused on intergroup contact with counter-stereotypical, high-status women, a boundary condition that satisfied Allport’s (1954) requirement of at-least-equal status between groups. However, a wealth of research since Allport (1954) has shown that contact can still reduce prejudice even in the absence of Allport’s suggested conditions (Brown and Hewstone 2005; Pettigrew and Tropp 2006), rendering these conditions optimal, but not necessary. In light of these findings, it is important to investigate the effects of contact with more stereotypical, lower-status women as well-something we did not do. There are at least two possibilities. Contact with more stereotypical women could have similar, although weaker effects, than contact with counter-stereotypical women. Conversely, in line with recent research on the negative effects of some kinds of contact (Barlow et al. 2012), contact with more stereotypical women could *increase* sexism. Future research that investigates these contrasting hypotheses directly would meaningfully increase our understanding of the effects of cross-gender contact.

We measured rape myth acceptance as a general variable, rather than examining specific types of rape myths. Although this measure was sufficient for the purposes of our study, future research could examine specific types of rape myths in more detail. Our outcome measure for Study 2 was projected sexual arousal, which is derived from the myth that rape is a sexual act, and not a violent one, and thus can be pleasurable for the victim. Future research could examine other consequences of rape myths that create misconceptions that are damaging to rape victims. One example is the myth of stranger rape (Abrams et al. 2003), that is, the belief that genuine rape consists of rape by a stranger and/or multiple assailants with use of explicit violence. Asking female participants about their sexual arousal concerning stranger rape specifically, for example, may elicit less favourable responses about rape than did the current study.

Both studies presented in this current research were correlational; hence no causal relationships can be determined from them. This is a shortcoming of all correlational research, and we acknowledge the possibility that the causal relationships may differ from those we hypothesised. For example, it is possible that men high in sexism are less likely to seek contact with counter-stereotypical women because they perceive these women as unpleasant or threatening. Furthermore, we recognise that the relationships between contact and sexism (and the manifestations of sexism) could be bi-directional. That said, the results of our study are in line with a wealth of previous research which shows that that intergroup contact indeed reduces prejudice (Pettigrew and Tropp 2006). Our study is useful in that it identified important new relationships among contact, sexism, rape myth acceptance, men’s intentions to rape, and women’s sexualisation of rape. However, future experimental research should be conducted to establish the causal relationships among these variables.

Finally, all the measures used in our study were self-reported and explicit, and we acknowledge that participants’ responses may have been influenced by demand characteristics. We took steps to address this possibility; participants were initially deceived about our hypotheses and we used filler items to further mask the true nature of the research. Still, some participants may have guessed the true hypothesis and modified their responses accordingly. Future research could take advantage of measures that circumvent self-presentation biases (see, Nosek et al. 2002) to more fully address the question of demand characteristics.

### Conclusions

Rape continues to be a serious and prevalent crime. Furthermore, the widespread acceptance of rape myths reduces the perceived seriousness of the crime, allowing perpetrators to be seen less negatively and victims to be seen more negatively. Intergroup contact has been shown to be one of the most effective prejudice-reducing mechanisms, reducing several types of prejudice towards various groups in various contexts. Our study is the first known to find support for the hypotheses that *intergroup* and *intragroup* contact may also reduce sexism as well as some of sexism’s more damaging manifestations: rape myth acceptance, men’s intentions to rape, and women’s sexualisation of rape. As such it opens the door to a potentially fruitful new avenue of research on contact and sexism and points to a potentially powerful means of reducing one of the most severe of all traumas.
